# The Interaction of Pesticides with Humin Fractions and Their Potential Impact on Non-Extractable Residue Formation

**DOI:** 10.3390/molecules28207146

**Published:** 2023-10-18

**Authors:** Aleksandra Ukalska-Jaruga, Romualda Bejger, Bożena Smreczak, Jerzy Weber, Lilla Mielnik, Maria Jerzykiewicz, Irmina Ćwieląg-Piasecka, Elżbieta Jamroz, Magdalena Debicka, Andrzej Kocowicz, Jakub Bekier

**Affiliations:** 1 Department of Soil Science Erosion and Land Protection, Institute of Soil Science and Plant Cultivation—State Research Institute, Czartoryskich 8, 24-100 Puławy, Poland; bozenas@iung.pulawy.pl; 2 Department of Bioengineering, West Pomeranian University of Technology in Szczecin, Papieża Pawła VI/3, 71-459 Szczecin, Poland; lilla.mielnik@zut.edu.pl; 3 Institute of Soil Science, Plant Nutrition and Environmental Protection, Wroclaw University of Environmental and Life Sciences, 50-375 Wroclaw, Poland; jerzyweber@gmail.com (J.W.); irmina.cwielag-piasecka@upwr.edu.pl (I.Ć.-P.); elzbieta.jamroz@upwr.edu.pl (E.J.); magdalena.debicka@upwr.edu.pl (M.D.); andrzej.kocowicz@upwr.edu.pl (A.K.); jakub.bekier@upwr.edu.pl (J.B.); 4 Faculty of Chemistry, University of Wroclaw, 50-137 Wroclaw, Poland; maria.jerzykiewicz@chem.uni.wroc.pl

**Keywords:** sorption, soil organic matter, NER, pendimethalin, cypermethrin, acetamiprid, metazachlor, flufenacet

## Abstract

The constant influx of pesticides into soils is a key environmental issue in terms of their potential retention in the soil, thus reducing their negative impact on the environment. Soil organic matter (SOM) is an important factor influencing the environmental fate of these substances. Therefore, the aim of this research was to assess the chemical behavior of pesticides (flufenacet, pendimethalin, α-cypermethrin, metazachlor, acetamiprid) toward stable soil humin fractions (HNs) as a main factor affecting the formation of non-extractable residues of agrochemicals in soil. This research was conducted as a batch experiment according to OECD Guideline 106. For this purpose, HNs were isolated from eight soils with different physicochemical properties (clay content = 16–47%, pH_KCl_ = 5.6–7.7, TOC = 13.3–49.7 g·kg^−1^, TN = 1.06–2.90 g·kg^−1^, TOC/TN = 11.4–13.7) to reflect the various processes of their formation. The extraction was carried out through the sequential separation of humic acids with 0.1 M NaOH, and then the digestion of the remaining mineral fraction with 10% HF/HCl. The pesticide concentrations were detected using GC-MS/MS. The pesticides were characterized based on the different sorption rates to HNs, according to the overall trend: metazachlor (95% of absorbed compound) > acetamiprid (94% of absorbed compound) > cypermethrin (63% of partitioning compound) > flufenacet (39% of partitioning compound) > pendimethalin (28% of partitioning compound). Cypermethrin and metazachlor exhibited the highest saturation dynamic, while the other agrochemicals were much more slowly attracted by the HNs. The obtained sorption kinetic data were congruous to the pseudo-first-order and pseudo-second-order models related to the surface adsorption and interparticle diffusion isotherm. The conducted research showed that the processes of pesticide sorption, apart from physicochemical phenomena, are also affected by the properties of the pollutants themselves (polarity, K_OC_) and the soil properties (SOM content, clay content, and pH_KCl_).

## 1. Introduction

Modern agricultural practices use a huge amount of pesticides to control insect pests, pathogens, and weeds. Many agrochemicals persist in the soil and plant systems for a long time and pose a risk of migration into drinking water sources and the food chain [1,2,3]. These agrochemicals often occur as a mixture of multiple compounds in the soil due to their simultaneous and/or subsequent seasonal applications. Popular plant-protection products differ in their chemical activity, disintegration time, and properties related to impacts on living organisms [4,5]. Among the various pesticide compounds, insecticides and herbicides are distinguished depending on their selective weed and pest control abilities [6]. Flufenacet, pendimethalin, and metazachlor are very common active substances due to their high effectiveness in combating specific groups of plants, without affecting the physiological processes of the crops. Similarly, acetamiprid and cypermethrin are widely used as insecticides in many commercial formulations. However, the EU Commission has withdrawn the approval for alpha-cypermethrin due to its carcinogenic properties with Regulation (EU) 2021/795, with effect from 17 May 2021. Nevertheless, commercial products containing this compound can still be exported from outside the EU, endangering the health of living organisms. The market of plant-protection products is still a challenge for researchers due to the emergence of new substances, as well as their behavior in the environment and interactions with other substances. This problem concerns, in particular, the soil environment, where the uncontrolled processes of the transformation of pesticides may occur due to the high heterogeneity of soil components.

The behavior of pesticides in soils is governed by a variety of complex dynamic physical, chemical, and biological processes, including sorption–desorption, volatilization, chemical and biological degradation, uptake by plants, run-off, and leaching [3,6]. These processes directly control the transport of pesticides within the soil profile or food [5]. For pesticides deposited in the top horizon of soil, the storage capacity and kinetics of sorption control the transport velocity of a compound, as well as the change in its bioavailability over time [2,5]. So, the retention of pesticides largely depends on the soil mineral and soil organic phase (SOM), which influences the bioavailability/bioaccessibility of these compounds [7,8,9,10,11,12,13]. Pesticide binding affinity is the attraction between a pesticide and soil particles with the significant influence of environmental factors (humidity, temperature, insolation, pressure, etc.). Pignatello et al. [8] reviewed the various binding mechanisms between pesticides and soil components, specifying the nature and strength of chemical intermolecular interactions. Hydrogen bonding, van der Waals forces, ligand exchange, and charge transfer complexes represent weak binding energies, whereas covalent linkages lead to the formation of chemically stable bonds [14,15,16,17]. In contrast to the weaker physico-chemical linkages, covalent bonding results in the incorporation of the compound into the structure of the sorbent molecule, and transforms these compounds to become its integral part. Nevertheless, the formation of a stable balance between attractive and repulsive forces of pesticide and soil component atoms are mainly the consequence of aging processes [11,14,16,18]. Attempts to analyze the behavior of pesticides in soil as a combination of interactions with separated soil constituents have been described in many publications [2,3,6,12,13,14,15]. The range of soil components is large, and the components interact with each other in complex ways. So far, SOM is considered to be the main soil factor influencing pesticide retention [2,3,6,12,13,14,15,16,17]. Nevertheless, to a large extent, this approach is still empirical and requires confirmation using accurate measurements.

The sorption of pesticides by SOM depends on its fractional composition and the proportions between individual fractions, such as fulvic acids, humic acids and humins (HNs), kerogen, bitumen, and black carbon [17]. Different SOM components vary remarkably in their chemical structure and composition, and therefore exhibit different sorption properties to pesticides [19]. HNs are more enriched in crosslinked, condensed structures, resulting in a higher sorption affinity for hydrophobic non-polar compounds [17,19]. So far, the structure of HNs has not been widely recognized due to the limited scientific data concerning analyses of their molecular composition. Nevertheless, it has been proved that HNs have the greatest impact on the accumulation and persistence of pesticides in soils [8,12]. It is assumed that the binding or entrapment processes of organic contaminants are controlled by ring aromatic units and long aliphatic chains inside the three-dimensional polymer structures of HNs [8,20,21]. The desorption and remobilization of pesticides from these sorption sites takes more time, causing a decrease in putative availability and toxicity due to non-extractable residue (NER) formation. It seems probable that a significant release of pesticides will only occur following the degradation or oxidation of the SOM matrix [7]. Radiocarbon dating provides estimates of the average annual turnover rates of HNs from 100 to 2400 years, which makes the created durable intermolecular interactions resistant to degradation over a very long period of time [7,22]. The environmental significance of NERs hinges on the extent to which they become indistinguishable from OM. If the turnover of SOM releases compounds that have been rendered environmentally innocuous and indistinguishable from SOM through repeated transformation, then the formation of bound residues can be legitimately regarded as an acceptable environmental remediation solution (Davenport et al., 2021). Currently, NER formation is a very important issue, because the immobilization of contaminants in the environment is one of the most important criteria in the international regulation of organic chemicals [1,6,23]. Besides being used for hazardous chemical substances, it is central to determining chemical exposure and the subsequent risk of pesticides to biota.

Therefore, the aim of this research was to assess the chemical affinity of selected pesticides (flufenacet, pendimethalin, α-cypermethrin, metazachlor, acetamiprid) to stable soil HNs as a main factor affecting the formation of the NERs of agrochemicals in soil. This research was carried out on HNs isolated from eight soils with different physicochemical properties to determine their ability to interact with pesticides. Moreover, pesticides with varied molecular structures and affinities to SOM were used in this study in order to evaluate the impact of their chemodiversity on the ongoing processes.

## 2. Results and Discussion

### 2.1. Dynamics of Pesticide Sorption to HNs

**Figure 1:**
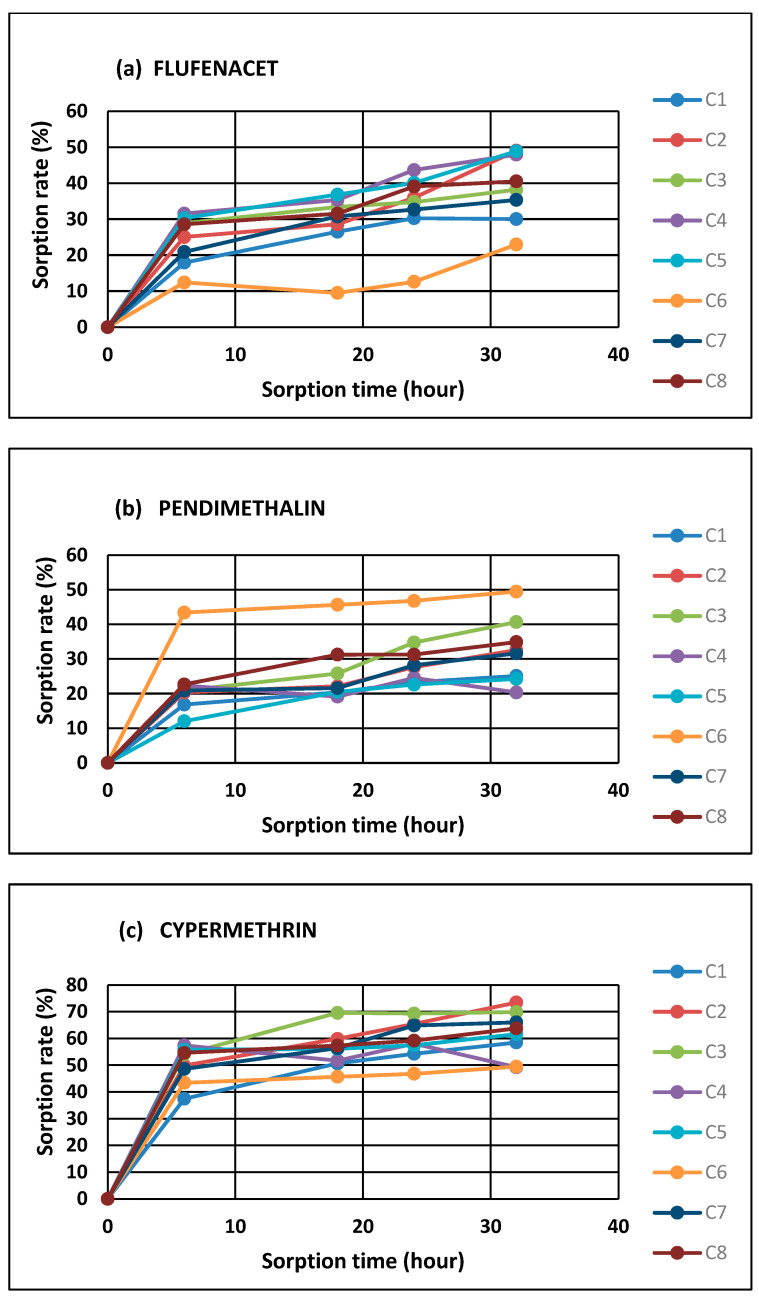

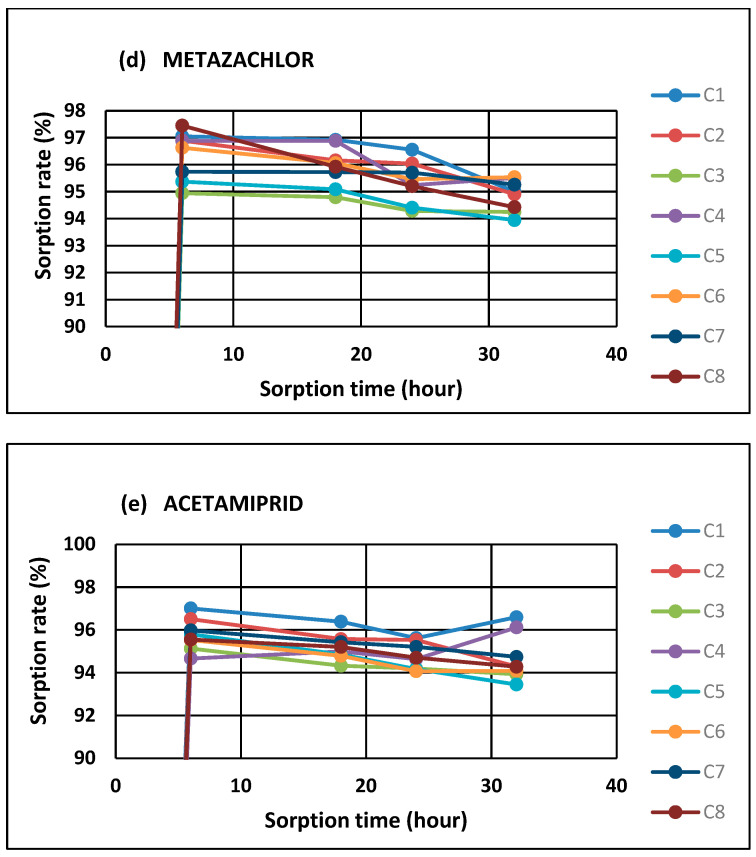
Kinetics of flufenacet (**a**), pendimethalin (**b**), cypermethrin (**c**), metazachlor (**d**), and acetamiprid (**e**) sorption to HNs (C1–C8—are HNs extracted from different soils).

The analyzed pesticides were characterized by differentiated sorption dynamics to HNs. The fastest sorption was observed in the first 6 h, and the contact time equilibrium during pesticide-HN contact reached after 32 h (Figure 1a–e; Table 1). Most of the compounds reached very rapid sorption in the first several hours, confirming the rapid saturation of the HN sorption sites. Metazachlor and acetamiprid exhibited higher saturation rates, while flufenacet, pendimethalin, and cypermethrin were much more slowly attracted by the HNs, with a significantly lower sorption efficiency (Figure 1a–e). Generally, the average pesticide capture amounts were observed in the following order: metazachlor (95% of absorbed compound) > acetamiprid (94% of absorbed compound) > cypermethrin (63% of absorbed compound) > flufenacet (39% of absorbed compound) > pendimethalin (28% of absorbed compound)—Table 1. The greatest differentiation in the sorption intensity was observed for flufenacet and pendimethalin (especially in the C6 sample of the HNs), but the general trend was maintained. Moreover, the concentration of metazachlor slightly decreased after 18 h by about 5%.

The observed isotherms are non-linear, presumably because there are many variously charged sites at each energy level, causing the distribution of the sorbent/adsorbent energies to not be a simple curve [24]. The obtained sorption kinetic data were congruous to the pseudo-first-order and pseudo-second-order models related to the surface adsorption and interparticle diffusion isotherm [25,26,27]. The most important consequence of isotherm calculations is that mobilities for chemicals at very high concentrations will be under-predicted values measured at lower concentrations, and vice versa. Thus, this effect may be amplified if several compounds are dissolved in the same test solution. Then, there is competition for sorption sites on the sorbent—selective adsorption—which most often indicates complex first- and second-order kinetics [24]. Despite the aforementioned factors, this is only an empirical model that approximately describes the processes potentially occurring at the interface of the HN phase and the pesticide solution phase; it allows us to explain in more detail the affinity of pesticides for HNs. According to these assumptions, chemisorption, as well as the physical penetration of HN structures by pesticides, may occur simultaneously. Thus, the surface of the HN is equipped with numerous selective sorption sites with a wide range of possible adsorption capacities for the pesticide molecules [25,26]. This allows for the creation of a multilayer and heterogeneous adsorption phase on the HN structure during the adsorption process. Moreover, the contaminants may occupy the stronger binding sites of the adsorbent first, and as the degree of occupation increases, the binding strength decreases [25,28]. The polynomial equation indicates that (variable ‘x’ less than 1—Table 1) the adsorption process is considered favorable, showing the formation of stronger interactions between the adsorbent and the adsorbate molecules [25,26,28], which was observed in our research (Table 1). This result was attributable to the higher specific surface area of HNs and the abundance of functional groups, which could contribute to pesticide strong binding. However, the most important conclusion drawn from the isotherm calculations is the under-prediction of chemical mobility at very high concentrations, and overprediction at very low concentrations, particularly pronounced in multi-component mixtures, as in the presented study. Then, there is the competition for sorption sites on the sorbent—selective adsorption—which most often indicates complex first- and second-order kinetics.

On the particle scale, apart from the processes of forming chemical bonds, sorption kinetics involve different physical processes related to the diffusion, e.g., sorption at the interface of soluble and insoluble organic matter and intraparticle diffusion to the sorbent structure [8,20,21,26]. It is assumed that the fast sorption kinetics observed in our study (Figure 1; Table 1) are mainly attributed to the dynamic trapping of pesticides through the external reactive moieties of the HNs (surface bonding) to then promote slow diffusion into the interior of the three-dimensional phase [20,21]. The diffusion limited the sorption/desorption kinetics by achieving the linearity of the sorption isotherm and reaching a complete sorption equilibrium before desorption starts again [18,26]. Therefore, the plot of sorption kinetics shows a plateau, provided that no structural changes to the sorbent occur during sorption/desorption. Overall, adsorption to HNs may encompass several qualitatively different processes: (1) the resting of molecules on discrete surface sites, (2) the partitioning of molecules into an ordered microscopic hydration phase near the surface, (3) the condensation of the pesticide molecules into a liquid-like state in small pores, and (4) the layering of molecules on the surfaces of water films that coat HNs [8,14]. Thus, it can be assumed that, in the conducted research, HNs during pesticide sorption undergo insignificant structural transformation (aggregation and disaggregation) processes, according to the theory of their polymer character [8,20,21].

The amorphous nature of HNs is reviewed with respect to the glassy, rubbery, and crystalline phases, which strongly differ in mobility, physical aging, inherent properties, density, and other characteristics which determine their chemical behavior [8,20,21,22,29]. HNs are mainly dominated by the presence of a condensed strong hydrophobic core with numerous aliphatic side chains [8,19,20,21,22], enabling different magnitudes of pesticide sorption. Polymeric particles of HNs may create micelles of a polymeric nature, where the basic structure consists of an aromatic ring (di- or trihydroxy phenyl type) bridged by -O-, -NH-, -N=, -S-, and other functional groups that contain both free -OH and -COOH groups and the double linkages of quinones, comparable to other SOM fractions [17,19,29]. The glassy phase forms regions of disordered side chains, which have a high density and low reactivity and flexibility, due to the presence of numerous unsaturated bonds [8,20,21,29] equipped with stable binding sites, which allow for the occlusion of other molecules in contrast to the rubbery phase [8,29]. Thus, HNs may react with non-ionic pesticides via hydrophobic retention as the main adsorption mechanism [12,13]. This may occur on aliphatic active sites, lipid constituents, and lignin-derived moieties with a high content of carbon and a small number of polar groups [30]. All of these functionalities may be involved in the adsorption of pesticides directly via van der Waals forces, hydrogen bonding, or indirectly by affecting the hydrophilic/hydrophobic balance of the HN surface [14,15]. All these processes can occur simultaneously, especially in the natural environment, with the additional influence of environmental factors and pesticide chemodiversity.

### 2.2. Impact of Individual Pesticide Properties on Sorption Rate to HNs

**Figure 2:**
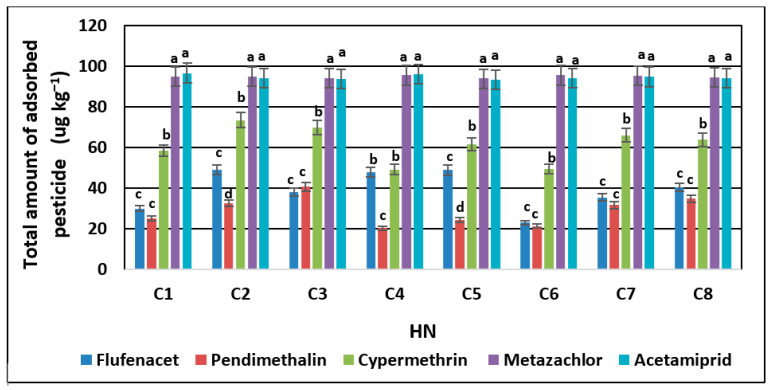
Total amounts of adsorbed pesticides on HNs (C1–C8). Small letters represent the result of the ANOVA aimed at evaluating the impact of variations in pesticide sorption on HNs.

The differences in sorption observed in the case of the tested pesticides undoubtedly result from the properties of these compounds and their affinity to HN structures (Figure 2). 

Generally, the obtained results indicated that metazachlor and acetamiprid were characterized by the highest sorption affinity compared to the other analyzed pesticides. The observed diversifications in the average values of the adsorbed compounds (metazachlor > acetamiprid > cypermethrin > flufenacet > pendimethalin) pointed out that the retention of pesticides by organic matter may be dependent on their polarity due to the high content of hydrophilic functional groups in the HN structures. The observed sorption relations were correlated with the polar surface areas of the pesticides (R = −0.95). The polar surface area is obtained by subtracting the area of carbon atoms, halogens, and hydrogen atoms bonded to carbon atoms (i.e., nonpolar hydrogen atoms) from the molecular surface. In other words, the polar surface area is the surface associated with heteroatoms (namely oxygen, nitrogen, and phosphorous atoms) and polar hydrogen atoms [31]. An extensive study of polar surface area values highlights that this parameter correlates better with hydrogen bonding (both donor and acceptor groups) than with lipophilicity [32]. This may indicate the dominance of hydrogen bonds and van der Waals interactions in the case of the analyzed pesticide–HN relations. Jing et al. [33] suggest that the sorption of polar pesticides by soil is due to the interaction between the –OH pharmacophore and the polar functional groups of humic substances, implying that hydrogen bonding may be the main sorption mechanism. This study highlighted the potential sorption mechanisms of pharmacophores in pesticide compounds with humic substances. Similar conclusions were drawn by Ćwieląg-Piasecka [13] in the research on the sorption of non-ionic pesticides in soils, with a varied content of stable forms of organic matter. Moreover, Kornilov et al. [32] underlined that the polar area is sensitive to spatial conformation for a given molecule, which confirms that the diffusion related to the partitioning of contaminants into the interior structures of organic matter molecules comprises very slow processes related to the reorganization and relaxation of the sorbent structures [8]. Diffusion can occur mainly in aging processes, in which the transport of pesticide molecules into the HN structure results from strong intermolecular interactions or processes of HN transformation during humification [17], hence the observed very fast sorption in the first phase of pesticide–HN contact, which then slowly stabilizes, reaching a plateau (Figure 1a–e). However, the conducted experiment does not allow us to clearly indicate which processes—equilibrium partitioning or sorption—dominate the observed relationships. For this purpose, it would be necessary to deepen the analysis of ongoing processes by using tools and techniques that allow for the observation of dynamic changes in HN structures over time.

Nevertheless, strong correlations were observed between the average sorption value and the Koc partition coefficient for the tested compounds (R^2^ = 0.68) determined for the dual isotherm model sorption, which describes adsorption on energetically heterogeneous surfaces and on microporous adsorbents. The organic carbon partition coefficient indicates a magnitude of pesticide affinity for the organic matter fraction. Its high values suggest that the pesticide is firmly retained, with a high potential for NER formation, which limits its migration and bioavailability in the soil. In the case of the analyzed compounds, the highest adsorption coefficient to the organic phase was shown by pendimethalin (K_foc_ = 13,792) and the lowest by metazachlor (K_foc_ = 79.6), which means that the stability of the interactions are inversely proportional to the rate of sorption. This is probably due to the fact that HNs are lipophilic forms of organic matter that are mainly rich in waxes, cutins, fats, bitumen, and other aliphatic chain compounds [19,22]. However, it should be kept in mind that the concept of the K_foc_ parameter is based on the assumption that the occurring interactions are non-polar, which means that they are subjected to a higher error when assessing the sorption of polar substances or in the case of low or high organic matter content affected by soil properties.

### 2.3. Impact of Soil Properties on Pesticide Sorption Rate to HNs

**Figure 3:**
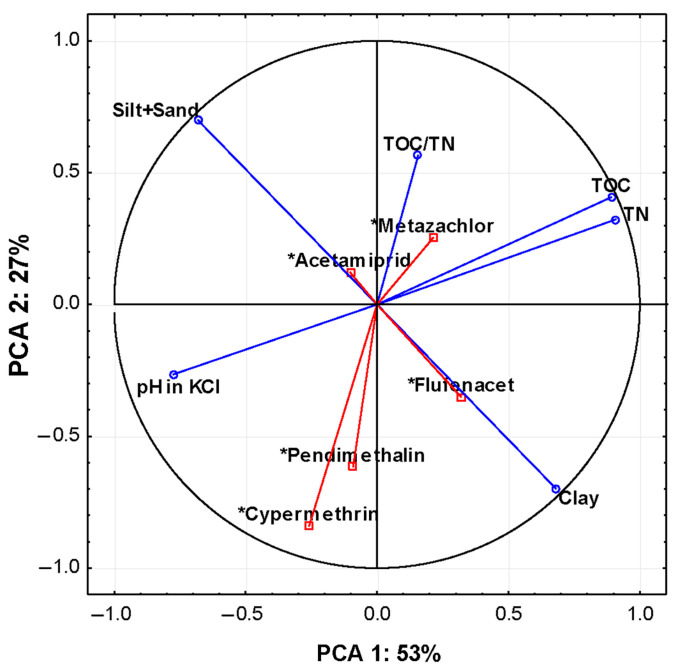
Principal component analysis (PCA) ordination biplot (PCA 1 vs. PCA 2); eigenvectors of the correlation matrix used to generate PCA components representing the impact of soil properties on pesticide sorption to HNs for all data sets (*n* = 8). Arrows/lines in biplot represent variable loadings relative to each component.

As presented in the previous chapters, HNs affect the sorption of pesticides in different ways, which may be related to the properties of the analyzed soils corresponding to the dominant HN formation process (Table 1). Therefore, a PCA was carried out to define a particular relationship between the sorption of pesticides to HNs and the types of analyzed soils (Figure 3, Table 2). The PCA analysis showed that 96% of the variability of the sorption of individual pesticides is explained by the first three PCA factors. The first PCA component (PCA 1), which accounted for 53% of variance, was significantly correlated with soil properties, i.e., pH (R^2^ = 0.68), TOC (R = −0.74), and TN (R = −0.74), which indicate their greatest impact on the accumulation potential of pendimethalin and cypermethrin (R^2^ = 0.80 and R^2^ = 0.94, respectively). The second PCA component (PCA 2), which represented merely 27% of the flufenacet sorption variance, was significantly correlated with TN (R = 0.63), TOC (R = 0.58), pH (R = −0.56), and the particle size distribution (R = 0.89 for clay and R = −0.89 for silt and sand), while the third component (PCA 3) was related to the concentration of the acetamiprid (R = 0.69) and TOC/TN ratio (R = 0.90) and accounted for 15% of the total variance. 

The obtained results indicated that the quantitative content of SOM and related parameters, including TN and TOC/TN, affect mainly the sorption potential of flufenacet, pendimethalin, metazachlor, and cypermethrin to HNs. These pesticides are characterized by the lowest value of HN sorption, which means that they probably have a higher affinity for more soluble forms and hydrophilic moieties of organic matter present in, e.g., fulvic or humic acids. 

The diversity of the structure of SOM determines the sorption of pesticides in accordance with the dual-model sorption concept [26,27]. Within this theory, SOM is assumed to be composed of two domains, with one displaying linear and non-competitive absorption or partitioning (non-carbonized organic matter) and the other showing non-linear, extensive and competitive surface adsorption (carbonized organic matter) depending on the type of fraction (fulvic acids, humic acids). During humification, individual domains are transformed, which means that pesticide molecules can combine with SOM, including HNs, to a different extent. It has been proved that pesticides can play the role of an abiotic catalyst in oxidative coupling processes occurring during the humification of organic matter [34]. These processes are catalyzed by the participation of microorganisms, which mediate both NER pesticide formation and its degradation [1,4,6]. Additionally, the pesticides which are most likely to bind covalently to SOM have functionalities similar to their components, which may explain our observations.

In addition, the obtained data showed that the particle size distribution also has an important impact on pesticide sorption to HNs, which may be due to the high share of the clay fraction in the formation of SOM structures. The contribution of clay in SOM stabilization supports its humification processes, and thus the formation of stable HN forms. Moreover, clay constitutes charged particles that have strong electrostatic properties capable of attracting pesticides, depending on the charge value [31,32,33,34,35]. Li et al. [36] pointed out that the adsorption of nitroaromatic compounds can be dominated by strongly hydrated cations in clay materials, e.g., sodium or calcium ions. Some other studies reported by Ćwielag-Piasecka et al. [37] showed that goethite and montmorillonite were the major soil constituents responsible for pesticide retention in loamy sand soil with very low levels of total carbon. Moreover, Infante et al. [38] proved that montmorillonite (Mt), like other smectites, as well as vermiculite (Vt), have permanent negative charges generated by the isomorphic substitutions of Si^4+^ by Al^3+^ and Mg^2+^, which maintain the interlayer surface charge. These permanent negative charges interact with cationic and neutral pesticides [39]. Al^3+^, Fe^3+^, Cr^3+^, Ti^4+^, and Zr^3+^ exchange with the interlayer cations and form polynuclear cationic species under hydrolysis, increasing the affinity towards anionic pesticide species [38]. In turn, Murano et al. investigated that mineral particles containing Al^3+^ and Fe^3+^ ions individually play only a minor role in the sorption process, but in interaction with humic substances, strongly enhance the level of pesticide sorption (in the case of an acetamiprid study) [40].

Ćwielag-Piasecka [13], as well as Clausen et al. [41], pointed out that pH is also an important factor that can significantly modify the retention of non-ionic pesticides, affecting the durability of the organic matter–pesticide bonds. This is partly because of their acid–base equilibria [24], but also because of the effects of pH on other SOM properties, such as electric charge and ionic strength [38]. 

## 3. Materials and Methods

### 3.1. Soil Sample Collection and Preparation

**Figure 4:**
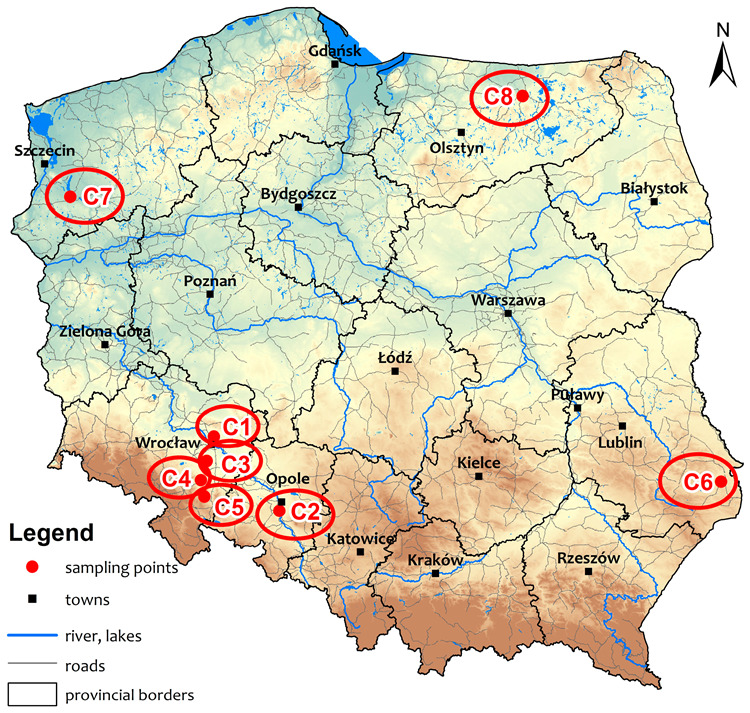
Location of sampling points selected for this study (*n* = 8).

The eight soil samples, characterized by different physicochemical properties, were selected for this study in order to precisely specify the determination of HNs (C1–C8) and their interactions with pesticides. The soils were sampled from 0 to 30 cm of chernozem and phaeozem, located in various agroecological zones of Poland (Figure 4).

The samples were prepared via drying and sieving and then analyzed for the particle size distribution, pH, total carbon, and total organic carbon (TC, TOC), as well as the total nitrogen concentration (TN). The particle size distribution was analyzed using the aerometric method (PN-R-04032, 1998) to accordingly determine the soil horizon texture, while the pH was measured potentiometrically in a 1:2.5 (m V^−1^) soil suspension in KCl (PN-ISO10390, 1997). The TOC and TN were analyzed using the dry combustion method on the TC/TN Vario Macro-Cube Elementar analyzer to express their mutual proportions, determining the initial degree of organic matter transformation in the selected soil samples. All measured soil parameters are included in Table 3.

### 3.2. HN Isolation

The detailed isolation procedure of the HNs was described in a previous methodological publication [42]. Briefly, the applied method is based on the classic separation of humic substances by eliminating alkali-soluble organic matter fractions (low molecular humic substances, fulvic acids, and humic acids) from the soil matrix via an exhaustive 0.1 M NaOH extraction. The remaining fraction was digested with a 10% HF/HCl mixture until the complete decomposition of the soil mineral forms. The collected HNs were lyophilized and then subjected to sorption laboratory experiments with pesticides. 

### 3.3. Pesticide Characterization and Method of Their Detection

To assess the interactions expressed based on the sorption affinity of pesticides to HNs, five active compounds of pesticides were selected: flufenacet, pendimethalin, cypermethrin, metazachlor, and acetamiprid. These compounds belong to a widespread group of toxic compounds and have high levels of use in agriculture as herbicides and insecticides. Their diversified physicochemical properties could affect their different behavior and persistence in soil due to variations in their molecular structure and binding potential based on stable organic matter forms (Table 4).

The determinations of the pesticide compounds were carried out using gas chromatography/triple mass spectrometry (GC-MS/MS; Agilent 7890B GC system; Agilent Tech., Santa Clara, CA, USA), equipped with an Agilent 7000C detector and Agilent 7693 Autosampler. The GC and MS/MS method parameters included a carrier gas flow at 1.00 mL min^−1^ (column 1) and 1.20 mL min^−1^ (column 2), an oven program (70 °C for 2 min at 25 °C min^−1^ to 150 °C for 0 min; 3 °C min^−1^ to 200 °C for 0 min; 8 °C min^−1^ to 280 °C for 10 min hold time), backflush settings for 5 min during post-run/310 °C, an inlet pressure of ~2 psi, a column pressure of ~3 psi, electron energy at 70 eV, a source temperature at 300 °C, and quad temperatures at 150 °C.

The GC was configured with a multimode inlet (MMI) equipped with a 4 mm ultra-inert, splitless, single-taper, glass wool liner (p/n 5190-2293). From the inlet, 2 HP-5ms UI columns (0.7 m × 150 μm, p/n 160-2625-5; and 30 m × 250 μm × 0.25 μm, p/n 19091S-431 UI, Agilent Technologies) were coupled with each other through a purged ultimate union for the use of mid-column/post-run backflushing.

The identification of pesticide compounds was performed in multiple reactor monitoring (MRM) mode with individual diagnostic ions. The quality of the determinations was controlled by the addition of a surrogate standard (PCB 155 compound: 2,2′,4,4′,6,6′-hexachlorobiphenyl, Dr. Ehrenstorfer GmbH, Augsburg, Germany), as well as an internal standard (PCB 207: 2,2′,3,3′,4,4′,5,6,6′-nonachlorobiphenyl, Dr. Ehrenstorfer GmbH, Augsburg, Germany) to control the analytical procedure and instrument indications.

The precision of the method was in the range of 1–6% (respectively, for flufenacet = 4.2%, pendimethalin = 1.2%, acetamiprid = 3.4%, metazachlor = 5.6%, cypermethrin = 4.7%) with the recovery within 92–97% (respectively, for flufenacet = 95.3%, pendimethalin = 93.4%, acetamiprid = 94.1%, metazachlor = 96.6%, cypermethrin = 92.3%) of the reference solution. The limit of detection (LoD) for each individual pesticide was at the 0.01 µg·kg^−1^ level. The detection limit value was adopted as the minimal value of the content of measured pesticides.

### 3.4. Sorption Experiments

The sorption experiment was carried out according to the OECD Guidelines for the Testing of Chemicals, no. 106, with five interval points (0, 6, 18, 24, and 32 h) in darkness and constant temperature conditions (20 ± 1 °C). The individual pesticides were dissolved in hexane (test solution) and added to isolated HNs at a concentration level of 10 µg·mL^−1^, e.g., 20 mL of pesticide solution and 0.5 g of HN. The liquid-to-solid proportion was established based on the Koc coefficient of the pesticides, according to the various percentages of adsorption (Figure 1 in OECD guideline 106). The obtained mixture enabled the sorption process at the interface between in the liquid–solid phase system, ensuring the free diffusion of compounds at the liquid–liquid interface, striving to achieve a state of dynamic equilibrium through the sorption of pesticides to HNs. Moreover, all of the fixed parameters were also evaluated, with the assumption that the concentration of the test compound in the solution should not exceed half of its solubility and be at least twice higher than the limit of detection of the GC MS/MS. The experiment was conducted in three replications for each individual compound with blank samples, such as reagent and matrix blanks, to ensure quality control processes.

Quantitative assessments of the pesticide concentrations adsorbed on the HNs were determined through GC MS/MS in the test-spiked solution after each period of time (0, 6, 12, 24, and 32 h). The adsorbed amount of the pesticides was calculated from the difference between the initial concentration in the test solution (control sample) and the concentration after a specified time.

### 3.5. Statistical Analysis

The software package Statistica (Dell Statistica, version 13.3) was used for statistical analysis. Basic statistical parameters such as the mean and standard deviation were calculated based on the results to detect gross errors in the performed analysis. Spearman’s correlation was used to assess the strength of the dependence of the pesticides’ properties on their amount of sorption to HNs over time, with the description based on an exponential function according to the assumption of fit to the pseudo-first-order and pseudo-second-order equation models [25]. The one-way analysis of variance (ANOVA) with a Mann–Whitney U test was used to evaluate the difference between the sorption affinities of individual pesticides to HNs. The principle component analysis (PCA) was used to evaluate the influence of soil properties on HN sorption potential in relation to pesticides. The PCA was based on the determination of factors that indicate a strong correlation between soil properties and pesticide sorption values. The obtained PCA data allowed us to determine which soils may be characterized by a higher HN sorption capacity in relation to agro-contaminants.

## 4. Conclusions

The obtained results indicated that pesticides exhibited different sorption potentials to HNs, which affect the formation of NERs. Generally, the total pesticide binding amount followed the order of metazachlor > acetamiprid > cypermethrin > flufenacet > pendimethalin, with the greatest variation in the sorption intensity observed for flufenacet and pendimethalin. The sorption kinetic data were nonlinear and congruous to the pseudo-first-order and pseudo-second-order models, related to the interparticle adsorption/diffusion isotherm. This proves that the surfaces of the HNs are equipped with numerous selective sorption sites with a wide range of possible adsorption capacities for pesticides. Additionally, the diversifications in the average values of the adsorbed compounds pointed out that the retention of pesticides by HNs may increase with decreases in their polarity, e.g., the sorption rate and value decreased with the increasing polar surface area of the pesticides. The observed relations indicate the dominance of hydrogen bonds and van der Waals interactions in the occurring sorption processes, as well as diffusion into the internal structures, according to the pseudo-first-order equation. Nevertheless, these phenomena occur mainly as a result of the aging time of pesticides in soils.

Moreover, this research pointed out that soil properties, apart from the agro-contaminant properties, may also influence the sorption of some compounds. Thus, the binding of pendimethalin, flufenacet, cypermethrin, and metazachlor was strongly dependent on the pH, clay minerals, and SOM content, as well as the TOC/TN ratio to the greatest extent, while acetamiprid was the least affected by these parameters. The significant influence of adsorbate properties on the HN–pesticide interactions directly result from its chemical affinity to the adsorbent, whereas the significance of soil properties on the occurring processes results from their direct impact on the formation and stabilization of HNs in the soil.

## Figures and Tables

**Table 1:** Total amount (% of initial concentration) and sorption equation of pesticides to HNs.

	Sorption Equation	Total Amount of Adsorbed Pesticide (Mean ± sd)
Flufenacet		
C1	y = −0.04x^2^ + 2.2x + 2.1	30.0 ± 1.2
C2	y = −0.01x^2^ + 1.7x + 5.5	49.1 ± 0.9
C3	y = −0.06x^2^ + 2.7x + 5.3	38.2 ± 1.1
C4	y = −0.05x^2^ + 2.8x + 5.9	48.0 ± 1.5
C5	y = −0.05x^2^ + 2.7x + 5.8	48.9 ± 0.8
C6	y = 0.01x^2^ + 0.3x + 3.8	23.0 ± 0.9
C7	y = −0.05x^2^ + 2.5x + 2.9	35.4 ± 1.4
C8	y = −0.05x^2^ + 2.6x + 5.1	40.5 ± 1.6
Pendimethalin		
C1	y = −0.03x^2^ + 1.6x + 2.8	25.1 ± 1.2
C2	y = −0.03x^2^ + 1.6x + 4.0	32.6 ± 1.7
C3	y = −0.02x^2^ + 1.8x + 3.7	40.7 ± 1.5
C4	y = −0.05x^2^ + 1.9x + 4.3	20.3 ± 1.3
C5	y = −0.03x^2^ + 1.6x + 1.2	24.2 ± 0.9
C6	y = −0.08x^2^ + 3.9x + 8.4	21.4 ± 1.5
C7	y = −0.03x^2^ + 1.7x + 4.2	31.6 ± 1.4
C8	y = −0.05x^2^ + 2.5x + 3.6	34.9 ± 1.3
Cypermethrin		
C1	y = −0.08x^2^ + 4.0x + 5.7	58.5 ± 1.3
C2	y = −0.09x^2^ + 4.7x + 8.9	73.5 ± 1.2
C3	y = −0.13x^2^ + 5.9x + 8.5	69.9 ± 1.5
C4	y = −0.13x^2^ + 5.1x + 11.1	49.2 ± 1.1
C5	y = −0.10x^2^ + 4.7x + 11.3	61.6 ± 1.6
C6	y = −0.08x^2^ + 3.8x + 8.5	49.5 ± 1.0
C7	y = −0.09x^2^ + 4.8x + 8.3	66.0 ± 0.9
C8	y = −0.10x^2^ + 4.8x + 10.8	63.8 ± 0.9
Metazachlor		
C1	y = −0.21x^2^ + 8.7x + 19.1	95.0 ± 1.1
C2	y = −0.20x^2^ + 8.6x + 19.1	94.9 ± 1.6
C3	y = −0.19x^2^ + 8.4x + 18.8	94.2 ± 1.7
C4	y = −0.20x^2^ + 8.5x + 19.2	95.5 ± 1.0
C5	y = −0.19x^2^ + 8.4x + 18.8	93.9 ± 1.5
C6	y = −0.20x^2^ + 8.5x + 19.2	95.5 ± 1.4
C7	y = −0.19x^2^ + 8.5x + 18.9	95.3 ± 1.6
C8	y = −0.20x^2^ + 8.5x + 19.4	94.4 ± 1.8
Acetamiprid		
C1	y = −0.19x^2^ + 8.4x + 19.4	96.6 ± 0.9
C2	y = −0.20x^2^ + 8.5x + 19.1	94.3 ± 0.8
C3	y = −0.19x^2^ + 8.3x + 18.9	93.9 ± 1.0
C4	y = −0.19x^2^ + 8.3x + 18.8	96.1 ± 1.1
C5	y = −0.20x^2^ + 8.4x + 19.0	93.4 ± 1.2
C6	y = −0.19x^2^ + 8.3x + 19.0	94.1 ± 0.9
C7	y = −0.20x^2^ + 8.4x + 19.0	94.7 ± 0.9
C8	y = −0.19x^2^ + 8.4x + 18.9	94.3 ± 1.3

**Table 2:** Principal component analysis (PCA) factor loading matrix; loading ≥ 0.5 in bold (*n* = 8).

	PCA 1	PCA 2	PCA 3
Clay	0.22	**0.89**	−0.05
Silt + Sand	−0.22	**−0.89**	0.05
pH in KCl	**0.65**	**−0.56**	0.25
TOC	**−0.74**	**0.58**	0.30
TN	**−0.74**	**0.63**	0.12
TOC/TN	−0.23	−0.05	**0.90**
Flufenacet	0.20	**0.59**	−0.43
Pendimethalin	**0.80**	0.31	0.12
Cypermethrin	**0.94**	0.26	−0.17
Metazachlor	**−0.70**	−0.16	−0.35
Acetamiprid	−0.43	−0.35	**−0.65**
Eigenvalues	**3**	**2**	**1**
Total variance	**53**	**27**	**15**
Cumulative %	**53**	**80**	**96**

Clay particle fraction < 0.002 mm, silt + sand particle fraction > 0.002 mm, TOC—total organic carbon, TN—total nitrogen, TOC/TN—proportion between total organic carbon content and total nitrogen.

**Table 3:** Soil physicochemical properties (*n* = 8, C1–C8: number of soil samples selected for the study).

Soil Samples	Clay (%)	Sand + Silt (%)	pH in KCl	TOC (g kg^−1^)	TN (g kg^−1^)	TOC/TN
C1	16	84	7.71	13.3	1.06	12.5
C2	41	59	7.45	24.4	2.14	11.4
C3	22	78	7.52	21.2	1.60	13.2
C4	24	76	5.64	41.7	3.39	12.3
C5	19	81	7.39	26.1	2.03	12.8
C6	21	79	7.52	39.9	2.90	13.7
C7	24	76	7.48	24.6	2.12	11.6
C8	47	53	6.66	37.7	2.80	13.4

Clay particle fraction < 0.002 mm, silt + sand particle fraction > 0.002 mm, TOC—total organic carbon, TN—total nitrogen, TOC/TN—proportion between total organic carbon content and total nitrogen.

**Table 4:** Physical and chemical characterization of pesticides used in the laboratory experiment.

Properties	Flufenacet	Pendimethalin	Acetamiprid	Metazachlor	α-Cypermethrin
Chemical formula	C_14_H_13_F_4_N_3_O_2_S	C_13_H_19_N_3_O_4_	C_10_H_11_ClN_4_	C_14_H_16_ClN_3_O	C_22_H_19_Cl_2_NO_3_
Pesticide type	Herbicide	Herbicide	Insecticide	Herbicide	Insecticide
Substance group	Oxyacetamide	Dinitroaniline	Neonicotinoid	Chloroacetamide	Pyrethroid
Molecular mass (g mol^−1^)	363.33	281.31	222.67	277.75	416.30
Polar surface area (Å^2^)	83.6	104.0	52.3	38.1	59.3
Octanol–water partition coefficient at pH 7, 20 °C	P = 3.16 × 10^3^ Log P = 3.5	P = 2.51 × 10^5^ Log P = 5.4	P = 6.31 × 10^0^ Log P = 0.8	P = 3.09 × 10^2^ Log P = 2.49	P = 6.31 × 10^5^ Log P = 5.8
Vapor pressure at 20 °C (mPa)	0.09	3.34	1.73·10^−4^	0.093	0.00038
Henry’s law constant at 25 °C (Pa m^3^ mol^−1^)	1.3 × 10^−3^	1.27	5.30 × 10^−8^	5.9 × 10^−5^	5.30 × 10^−2^
Linear adsorption and mobility	K_oc_ = 401 mL·g^−1^	K_oc_ = 17,491 mL·g^−1^	K_oc_ = 200 mL·g^−1^	K_oc_ = 54.0 mL·g^−1^	K_oc_ = 288,735 mL·g^−1^
Freundlich adsorption and mobility	K_f_ = 4.38 K_foc_ = 273.3 1/*n* = 0.92	K_f_ = 220.1 K_foc_ = 13,792 1/*n* = 0.954	K_f_ = 1.58 K_foc_ = 106.5 1/*n* = 0.86	K_f_ = 1.02 K_foc_ = 79.6 1/*n* = 0.993	-
Bio-concentration factor (BCF, L kg^−1^)	71.4	5100	Low risk	Low risk	910
Threshold of Toxicological Concern (Cramer Class)	High (class III)	High (class III)	High (class III)	High (class III)	High (class III)

## Data Availability

All scientometric data generated during the study were deposited in the database of the authors and the projects under which they were generated. For more information, please contact aukalska@iung.pulawy.pl or romualda.bejger@zut.edu.pl.

## Sample Availability

All samples are available from the authors of the publication.
